# Serum N-terminal Pro-B-Type Natriuretic Peptide and Phosphodiesterase 4D Levels in Adults With Overt Hypothyroidism: A Case-Control Study

**DOI:** 10.7759/cureus.114452

**Published:** 2026-08-13

**Authors:** Syed Hasan Amir, Khwaja Saifullah Zafar, Ishan Sharma, Sheelu Shafiq Siddiqi

**Affiliations:** 1 Medicine, Jawaharlal Nehru Medical College Hospital, Aligarh, IND; 2 Diabetes and Endocrinology, Rajiv Gandhi Centre for Diabetes and Endocrinology, Jawaharlal Nehru Medical College Hospital, Aligarh, IND

**Keywords:** cardiovascular biomarkers, case-control study, cyclic nucleotide signalling, elisa, natriuretic peptides, nt-probnp, overt hypothyroidism, phosphodiesterase 4d (pde4d), subclinical cardiac dysfunction, thyroid and heart

## Abstract

Background: Thyroid hormone regulates cardiac and vascular function, and overt hypothyroidism predisposes to impaired ventricular relaxation, raised systemic vascular resistance, and heart failure with preserved ejection fraction. N-terminal pro-B-type natriuretic peptide (NT-proBNP) is a sensitive marker of myocardial wall stress and is itself under thyroid-hormone control, while cyclic-nucleotide phosphodiesterases (PDEs) shape the intracellular signalling that underlies myocardial contractility. The two have rarely been measured together in adults with overt hypothyroidism and seldom in India. We compared serum NT-proBNP and phosphodiesterase 4D (PDE4D), a cAMP-specific isoform, between adults with overt hypothyroidism and euthyroid controls.

Materials and methods: This hospital-based case-control study at a tertiary-care teaching hospital in north India was conducted with institutional ethics committee approval and written informed consent. We enrolled 80 adults (older than 20 years) in equal, age- and sex-matched overt hypothyroid and euthyroid groups (40 each). Blinded personnel measured serum triiodothyronine (T3), thyroxine (T4), thyroid-stimulating hormone (TSH), and NT-proBNP by chemiluminescent immunoassay and serum PDE4D by a sandwich enzyme-linked immunosorbent assay. Groups were compared with the independent-samples t-test or Mann-Whitney U test and the chi-square or Fisher's exact test, alongside univariable logistic regression and effect-size analysis (point-biserial r, Cramer's V); p < 0.05 was taken as significant.

Results: The groups matched closely for age (p = 0.617) and sex (72.5% female in each). Serum NT-proBNP ran about fourfold higher in the hypothyroid group (927.10 ± 487.47 vs 233.32 ± 126.12 pg/mL; p < 0.001; r = 0.70), while serum PDE4D ran about twofold lower (19.0 ± 5.9 vs 38.7 ± 9.7 ng/mL; p < 0.001; r = 0.78); PDE4D gave the highest Nagelkerke R² of any variable studied (0.884). The two markers thus moved in opposite directions. The hypothyroid group also carried higher BMI and blood pressure and more hypertension (35% vs 10%) and peripheral oedema (45% vs 20%).

Conclusions: In adults with long-standing overt hypothyroidism, serum NT-proBNP was markedly higher and serum PDE4D markedly lower than in euthyroid controls, a reciprocal pattern set against raised blood pressure, BMI, and hypertension. Cardiovascular surveillance in overt hypothyroidism may therefore be worthwhile, and serum PDE4D emerges as a promising, hypothesis-generating candidate marker that still needs prospective, multivariable-adjusted validation.

## Introduction

Thyroid hormone shapes almost every aspect of cardiovascular function, acting on the myocardium, the systemic vasculature, and the autonomic nervous system through both genomic and non-genomic pathways. Triiodothyronine drives the transcription of contractile and calcium-handling proteins, and in doing so, it sets myocardial contractility, relaxation, heart rate, and vascular resistance [1]. When thyroid hormone is deficient, these effects reverse. Overt hypothyroidism, defined biochemically by a raised serum thyroid-stimulating hormone (TSH) with a low free thyroxine, is one of the commonest endocrine disorders, and it brings impaired ventricular relaxation, higher systemic vascular resistance, a modest rise in diastolic pressure, and a tendency toward heart failure with preserved ejection fraction [2,3]. Detecting this cardiac involvement before frank failure develops is precisely why sensitive circulating markers have been sought for so long.

When a ventricular cardiomyocyte is stretched, it cleaves its prohormone into the active 32-amino-acid B-type natriuretic peptide (BNP) and the inactive 76-amino-acid N-terminal fragment, NT-proBNP. The N-terminal fragment lasts longer in the circulation and is more stable in vitro, which is why laboratories favour it as a marker of myocardial wall stress [4-6]; it tracks left-ventricular dysfunction, functional class, and prognosis in heart failure [7]. In thyroid disease, however, NT-proBNP reports more than haemodynamic load alone. Thyroid hormone itself switches on cardiac BNP transcription through response elements in the BNP promoter [8], so a hypothyroid patient's value cannot be read the same way as a euthyroid one. Hyperthyroidism reliably raises NT-proBNP; hypothyroidism does not behave so predictably, and the literature is genuinely mixed, with reports of reduced, unchanged, and even raised concentrations [9,10].

The second messengers cyclic adenosine monophosphate (cAMP) and cyclic guanosine monophosphate (cGMP) carry much of this signalling, and cyclic-nucleotide phosphodiesterases (PDEs) are the enzymes that break them down. By hydrolysing cAMP and cGMP in defined subcellular compartments, PDEs set where and for how long each signal acts, and so they help govern myocardial contractility, vascular tone, endothelial function, and cardiac remodelling [11-13]. Two families dominate cAMP breakdown in the cardiomyocyte, PDE3 and PDE4 (the latter including the cAMP-specific isoform PDE4D), and together they tune its β-adrenergic responsiveness. A different isoform, PDE8B, is abundant in the thyroid gland, where a common polymorphism (rs4704397) tracks circulating TSH in the general population [14]. Thyroid hormone alters the expression of several of these isoforms, which places thyroid status upstream of both cyclic-nucleotide signalling and natriuretic-peptide biology; measuring a marker from each axis may therefore say more about the cardiovascular cost of hypothyroidism than either alone [15].

PDE4D deserves particular attention among these enzymes. It sits within macromolecular complexes in the cardiomyocyte, close to the machinery it regulates, and there it sculpts the local cAMP gradients behind excitation-contraction coupling and the β-adrenergic response [11,12]. Deleting PDE4D in animal models is far from benign: the heart develops a progressive cardiomyopathy and fails early, a phenotype traced to protein kinase A hyperphosphorylation, destabilisation of the ryanodine-receptor (RyR2) calcium-release channel, and blunted β-adrenergic signalling [13,16]. PDE4D therefore sits at the meeting point of three thyroid-hormone-sensitive processes: cAMP signalling, calcium handling, and β-adrenergic tone. That location makes it plausible that the amount of PDE4D circulating in blood echoes disturbed cyclic-nucleotide signalling in the hypothyroid heart. Direct measurements of circulating PDE4D in hypothyroidism are almost non-existent, however, and that gap is what prompted us to measure it here.

Hypothyroidism is common the world over, with an estimated Indian prevalence of roughly 8-11% and a clear female predominance [17,18], and even its subclinical form has been tied to worse cardiovascular outcomes. Despite that, NT-proBNP and circulating PDEs have rarely been measured side by side in the same adults with overt hypothyroidism, and Indian data are scarcer still [19]. We therefore ran a hospital-based case-control study that compared serum NT-proBNP and serum PDE4D between adults with overt hypothyroidism and age- and sex-matched euthyroid controls. We expected both markers to depart from control values, and we hoped their pattern would help describe the cardiovascular biochemistry of the hypothyroid state.

## Materials and methods

Study design and setting

This was an observational, hospital-based case-control study conducted in the Department of Medicine and the Rajiv Gandhi Centre for Diabetes and Endocrinology, Jawaharlal Nehru Medical College and Hospital, Aligarh Muslim University, Aligarh, India, between December 2023 and June 2026. The study was designed and reported in accordance with the Strengthening the Reporting of Observational Studies in Epidemiology (STROBE) statement for case-control studies.

Participants

Adults older than 20 years were eligible. Cases had overt primary hypothyroidism, defined biochemically by an elevated serum TSH together with a reduced free thyroxine concentration on standard thyroid function testing and accompanied by consistent clinical features; both newly diagnosed patients and patients already receiving levothyroxine were included. Controls were euthyroid adults with biochemically normal thyroid function and no clinical evidence of thyroid disease, recruited from the same outpatient and inpatient services. Controls were frequency-matched to the cases for age and sex to minimise confounding of the biomarker comparisons. To limit alternative causes of natriuretic-peptide elevation, participants were excluded if they had established heart failure, coronary artery disease, or recent myocardial infarction, significant valvular heart disease, cardiomyopathy, or clinically significant arrhythmia, chronic kidney disease, chronic liver disease, chronic obstructive pulmonary disease, active infection or sepsis, or known malignancy. Pregnancy, age of 20 years or below, hyperthyroidism, haemolysed or otherwise contaminated samples, and unwillingness to participate were additional exclusion criteria.

Sample size

Eighty participants were enrolled and split equally into two groups of 40: an overt hypothyroid group and an age- and sex-matched euthyroid control group. Group size was governed mainly by how many eligible patients presented during the study window. For the primary biomarker comparisons, 40 per group gives about 80% power to detect a standardised between-group difference of 0.63 SD (Cohen's d), and more than 90% power for a large difference (d ≥ 0.73), at a two-sided alpha of 0.05; the effects actually observed for NT-proBNP and PDE4D comfortably exceeded those thresholds. Recruitment continued until each arm reached the planned number.

Data collection

Every participant underwent a standardised history and clinical examination. Age, sex, occupation, type and duration of hypothyroidism, current treatment and compliance, comorbidities, complications, anthropometry, and vital signs were recorded on a structured case-report form. Body mass index (BMI) was calculated and categorised using World Health Organization Asian-Indian cut-offs.

Laboratory methods

Venous blood was drawn under aseptic precautions. Serum total triiodothyronine (T3), total thyroxine (T4), TSH, and NT-proBNP were measured by chemiluminescent immunoassay. Serum PDE4D concentration was measured with a commercially available sandwich enzyme-linked immunosorbent assay (ELISA) specific for human PDE4D (human PDE4D ELISA kit, catalogue number EH3530; FineTest, Wuhan Fine Biotech Co., Ltd., Wuhan, China) used strictly according to the manufacturer's instructions. In brief, pre-coated anti-PDE4D microplates were incubated with samples and standards, followed by a biotinylated detection antibody, horseradish-peroxidase-conjugated streptavidin, and tetramethylbenzidine substrate, and optical density was read at 450 nm. The kit is calibrated against a recombinant human PDE4D standard over a working range of 78.125-5,000 pg/mL (analytical sensitivity 46.875 pg/mL). As serum concentrations exceeded the upper limit of this range, each sample was diluted so that its assayed value fell on the linear portion of the standard curve; the optical density was converted to concentration from a four-parameter-logistic fit and multiplied by the dilution factor to obtain the serum concentration. Serum PDE4D concentrations are reported in nanograms per millilitre (ng/mL; 1 ng/mL = 1,000 pg/mL); that is, the pg/mL value read from the standard curve was multiplied by the dilution factor and expressed in ng/mL. According to the manufacturer, the assay has intra-assay and inter-assay coefficients of variation below 10% (intra-assay: 4.4-5.3%; inter-assay: 4.7-5.1%). All assays were performed in batches by trained laboratory personnel who were blinded to the study-group allocation.

Statistical analysis

Data were analysed using Statistical Product and Service Solutions (SPSS, version 29.0; IBM SPSS Statistics for Windows, Armonk, NY). The distribution of continuous variables was assessed with the Shapiro-Wilk test. Normally distributed variables were compared using the independent-samples t-test and non-normally distributed variables using the Wilcoxon-Mann-Whitney U test; categorical variables were compared using the Pearson chi-square or Fisher's exact test. Continuous data are presented as mean ± standard deviation (SD) with median and interquartile range (IQR), and categorical data as frequency (percentage). Effect sizes were quantified using the point-biserial correlation coefficient (r) for continuous variables and the bias-corrected Cramer's V for categorical variables, interpreted using conventional thresholds (r ≥ 0.50 or V ≥ 0.50, large/high; 0.30-0.49, medium/moderate; 0.10-0.29, small/low). Univariable logistic regression with study group as the dependent variable yielded odds ratios (ORs) with 95% confidence intervals (CIs), the Nagelkerke pseudo-R², and the Hosmer-Lemeshow goodness-of-fit test; univariable linear regression yielded mean differences with 95% CIs. All tests were two-sided, and a p-value < 0.05 was considered statistically significant. To account for multiple comparisons across the variables that differed between groups, a Bonferroni-corrected threshold (p < 0.004 for 13 comparisons) was additionally considered; the two primary biomarker differences (both p < 0.001) remained significant under this stricter criterion, whereas some secondary associations did not. Regression analyses were univariable; because the thyroid analytes define case status, they produce near-complete separation in logistic models, and their ORs are therefore not reported.

Ethical considerations

The study was approved by the Institutional Ethics Committee of Jawaharlal Nehru Medical College, Aligarh Muslim University (approval no. IECJNMC/1308). Written informed consent was obtained from every participant in a language they understood, in accordance with the Declaration of Helsinki.

## Results

Baseline characteristics of the study population

The two study arms were demographically similar (Table 1). Mean age was 47.40 ± 13.41 years in the hypothyroid group and 45.55 ± 15.78 years in the euthyroid group (p = 0.617), and each arm held 29 women (72.5%) and 11 men (27.5%). Half of the hypothyroid cohort (50%) fell in the 40-60-year band, 67.5% had been diagnosed at least five years earlier, and 47.5% admitted poor compliance with levothyroxine. BMI was clearly higher in the hypothyroid arm (28.95 ± 1.76 vs 25.25 ± 3.43 kg/m^2^; p < 0.001), and not one hypothyroid participant had a BMI below 25 kg/m^2^. Blood pressure was higher too, both systolic (132.25 ± 20.52 vs 120.00 ± 12.00 mmHg; p = 0.003) and diastolic (84.97 ± 11.47 vs 78.10 ± 6.44 mmHg; p = 0.008), as were the prevalences of hypertension (35% vs 10%; OR: 5.92, 95% CI: 1.72-24.55; p = 0.008) and clinically apparent peripheral oedema (45% vs 20%; OR: 3.27, 95% CI: 1.21-8.84; p = 0.019). Heart rate, respiratory rate, diabetes prevalence, pallor, and icterus did not differ significantly between the groups.

**Table 1:** Baseline demographic, anthropometric, vital-sign, and clinical characteristics of the study population BMI: body mass index; BP: blood pressure; SD: standard deviation; SpO_2_: peripheral oxygen saturation Continuous variables were compared using the independent t-test or Wilcoxon-Mann-Whitney U test, and categorical variables using the chi-square or Fisher's exact test. *Statistically significant at p < 0.05.

Parameter	Euthyroid (n = 40)	Hypothyroid (n = 40)	p-value
Age, years, mean ± SD	45.55 ± 15.78	47.40 ± 13.41	0.617
Female, n (%)	29 (72.5)	29 (72.5)	1.000
Male, n (%)	11 (27.5)	11 (27.5)	–
BMI, kg/m², mean ± SD	25.25 ± 3.43	28.95 ± 1.76	< 0.001*
Systolic BP, mmHg, mean ± SD	120.00 ± 12.00	132.25 ± 20.52	0.003*
Diastolic BP, mmHg, mean ± SD	78.10 ± 6.44	84.97 ± 11.47	0.008*
Heart rate, beats/min, mean ± SD	78.55 ± 10.66	77.12 ± 9.05	0.897
SpO₂, %, mean ± SD	96.65 ± 1.87	97.40 ± 1.57	0.033*
Hypertension, n (%)	4 (10.0)	14 (35.0)	0.008*
Diabetes mellitus, n (%)	14 (35.0)	13 (32.5)	0.385
Peripheral oedema, n (%)	8 (20.0)	18 (45.0)	0.031*
On levothyroxine, n (%)	0 (0.0)	40 (100.0)	< 0.001*

Thyroid function profile

As the case definition dictated, the thyroid profile split the groups cleanly (Table 2). Serum T3 (0.50 ± 0.21 vs 1.36 ± 0.26 nmol/L) and T4 (4.94 ± 1.06 vs 9.33 ± 1.67 µg/dL) were far lower in the hypothyroid group, and TSH was more than tenfold higher (25.75 ± 9.71 vs 2.42 ± 1.13 mIU/L), all at p < 0.001; indeed, every TSH value in the hypothyroid arm exceeded every value in the euthyroid arm. That TSH stayed this high despite universal levothyroxine prescription confirms the cohort was still biochemically hypothyroid when sampled.

**Table 2:** Thyroid function profile by study group T3: triiodothyronine; T4: thyroxine; TSH: thyroid-stimulating hormone; CI: confidence interval Mean difference is euthyroid minus hypothyroid. Data are mean ± standard deviation. *Statistically significant at p < 0.05.

Thyroid parameter	Euthyroid (n = 40)	Hypothyroid (n = 40)	Mean difference (95% CI)	p-value
Total T3, nmol/L	1.36 ± 0.26	0.50 ± 0.21	0.86 (0.76 to 0.96)	< 0.001*
Total T4, µg/dL	9.33 ± 1.67	4.94 ± 1.06	4.39 (3.76 to 5.01)	< 0.001*
TSH, mIU/L	2.42 ± 1.13	25.75 ± 9.71	−23.33 (−26.45 to −20.21)	< 0.001*

Primary biomarkers: NT-proBNP and phosphodiesterase 4D

**Figure 1:**
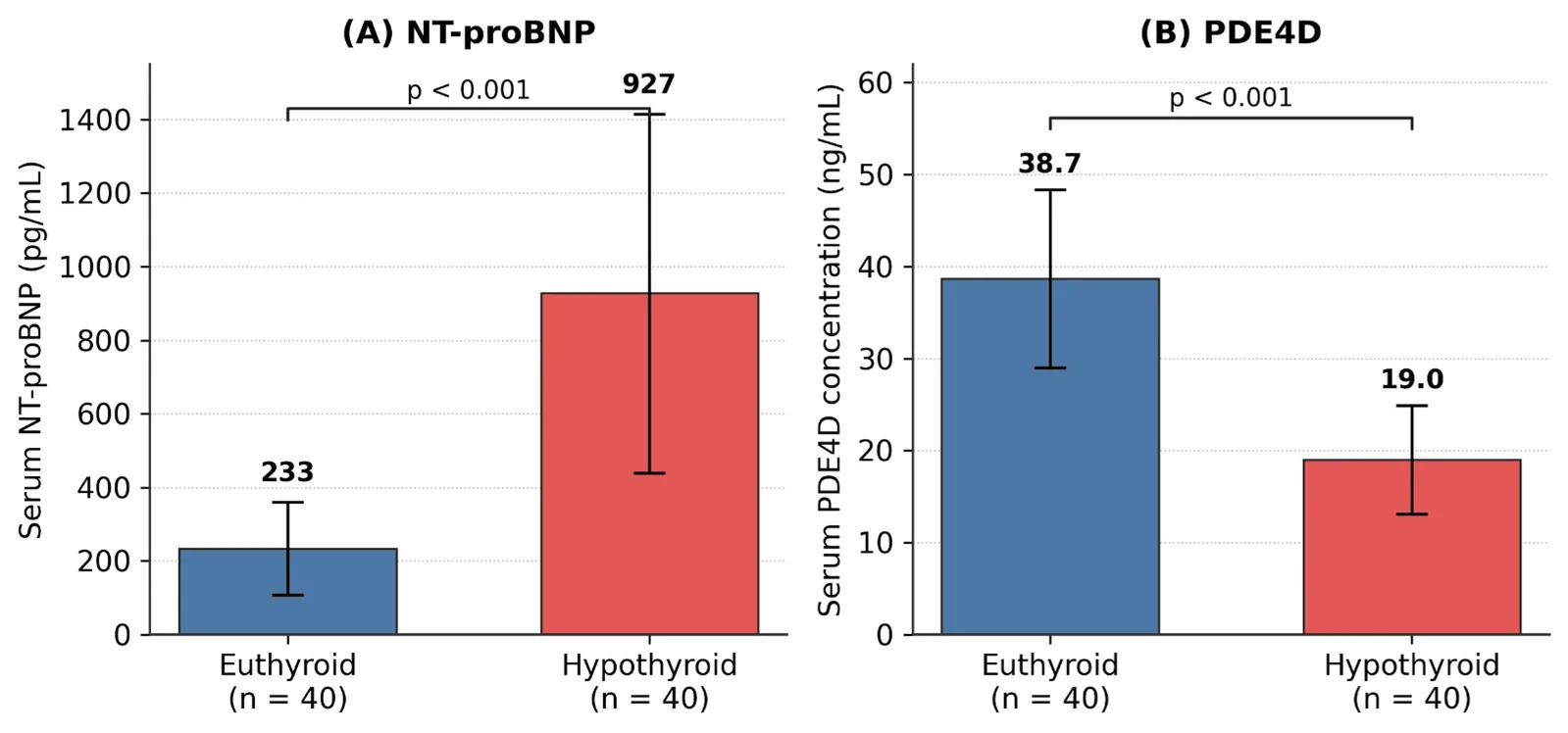
Mean serum NT-proBNP (panel A) and serum phosphodiesterase 4D (PDE4D) concentrations (panel B) in the euthyroid and overt hypothyroid groups Bars show the group mean and error bars show ± 1 standard deviation. NT-proBNP was approximately four-fold higher and PDE4D concentration approximately two-fold lower in the hypothyroid group (both p < 0.001). NT-proBNP: N-terminal pro-B-type natriuretic peptide; PDE4D: phosphodiesterase 4D.

Serum NT-proBNP was roughly four-fold higher in the hypothyroid group (927.10 ± 487.47 vs 233.32 ± 126.12 pg/mL; median: 938.00 vs 238.00 pg/mL; p < 0.001), a large effect (r = 0.70). Serum PDE4D ran the other way, roughly two-fold lower (19.0 ± 5.9 vs 38.7 ± 9.7 ng/mL; median: 19.5 vs 37.0 ng/mL; p < 0.001; r = 0.78). The two markers therefore diverged in the hypothyroid state (Table 3, Figure 1).

**Table 3:** Serum NT-proBNP and phosphodiesterase 4D concentration by study group NT-proBNP: N-terminal pro-B-type natriuretic peptide; PDE4D: phosphodiesterase 4D; SD: standard deviation; IQR: interquartile range Between-group comparisons used the Wilcoxon-Mann-Whitney U test. *Statistically significant at p < 0.05.

Biomarker	Euthyroid (n = 40)	Hypothyroid (n = 40)	Point-biserial r	p-value
NT-proBNP, pg/mL, mean ± SD	233.32 ± 126.12	927.10 ± 487.47	0.70 (large)	< 0.001*
NT-proBNP, pg/mL, median (IQR)	238.00 (112.75-347.00)	938.00 (608.00-1099.75)	–	–
PDE4D, ng/mL, mean ± SD	38.7 ± 9.7	19.0 ± 5.9	0.78 (large)	< 0.001*
PDE4D, ng/mL, median (IQR)	37.0 (33.0-41.8)	19.5 (14.0-24.0)	–	–

Univariable regression and effect-size synthesis

**Figure 2:**
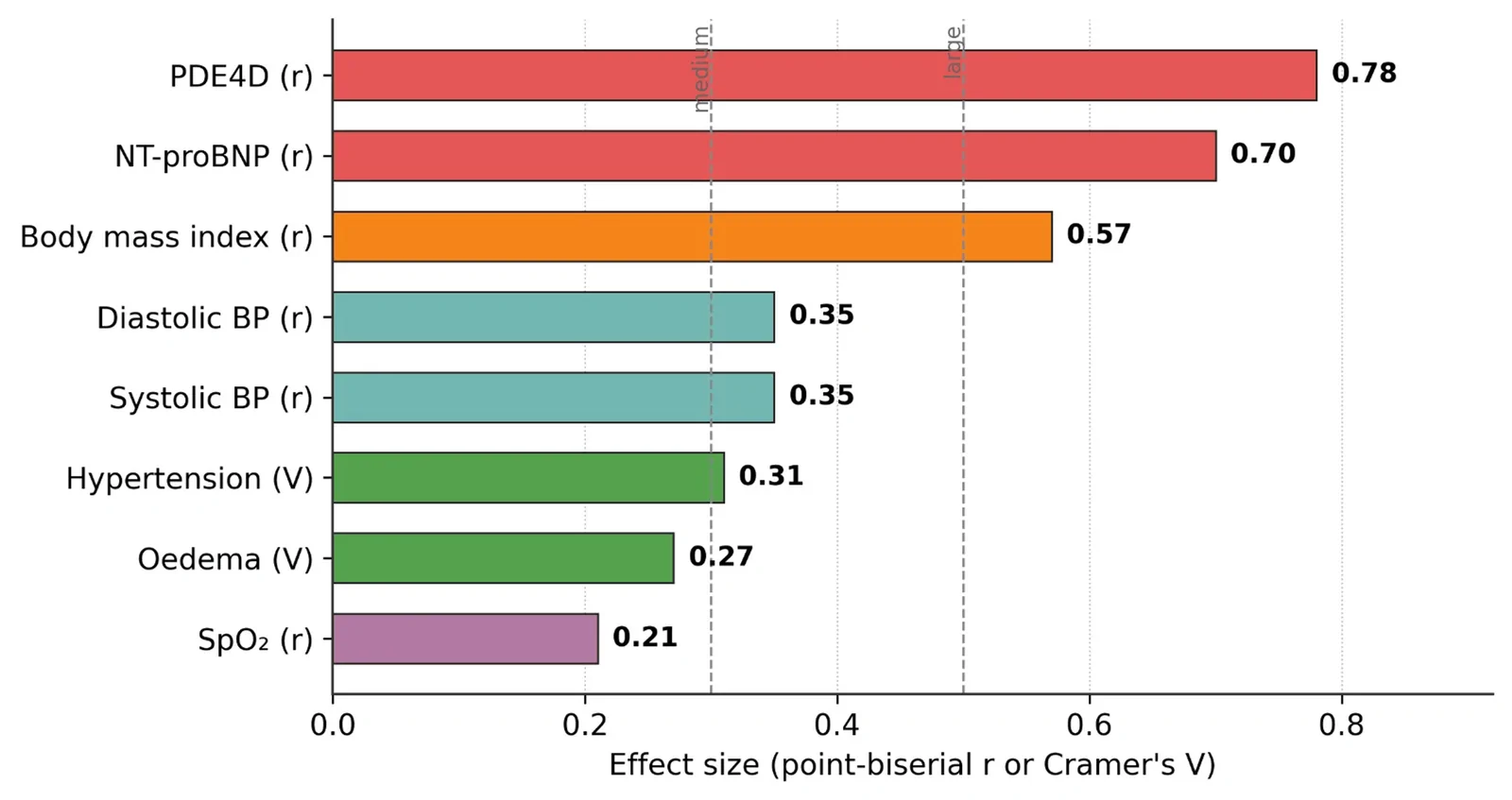
Effect sizes (point-biserial r for continuous variables, Cramer's V for categorical variables) for all parameters that differed significantly between the euthyroid and hypothyroid groups, excluding the case-defining thyroid analytes BP: blood pressure; NT-proBNP: N-terminal pro-B-type natriuretic peptide; PDE4D: phosphodiesterase 4D Dashed lines mark the conventional thresholds for medium (0.30) and large (0.50) effects. PDE4D and NT-proBNP show the largest effects.

On univariable logistic regression with study group as the outcome (Table 4, Figure 2), PDE4D discriminated the groups most strongly (OR: 0.646 per ng/mL, 95% CI: 0.484-0.770; Nagelkerke pseudo-R² = 0.884; Hosmer-Lemeshow p = 0.942), followed by NT-proBNP (OR: 1.007 per pg/mL, 95% CI: 1.005-1.012; Nagelkerke pseudo-R² = 0.692; Hosmer-Lemeshow p = 0.652) and BMI (OR: 1.674 per kg/m²; Nagelkerke pseudo-R² = 0.425). Ranking every significant between-group difference by magnitude told the same story: after the case-defining thyroid analytes, the two study biomarkers carried the largest effects, with PDE4D (r = 0.78) and NT-proBNP (r = 0.70) outstripping BMI (r = 0.57), the blood-pressure measures (r = 0.35), and the categorical clinical findings (Figure 2).

**Table 4:** Univariable logistic regression and effect sizes for variables differing significantly between the groups OR: odds ratio; CI: confidence interval; BMI: body mass index; BP: blood pressure; NT-proBNP: N-terminal pro-B-type natriuretic peptide; PDE4D: phosphodiesterase 4D; r: point-biserial correlation coefficient; V: Cramer's V The odds ratio for PDE4D is below 1 because higher PDE4D concentrations are associated with euthyroid status. *Statistically significant at p < 0.05.

Variable	OR (95% CI)	Nagelkerke R²	Effect size	p-value
PDE4D (per ng/mL)	0.646 (0.484-0.770)	0.884	r = 0.78	< 0.001*
NT-proBNP (per pg/mL)	1.007 (1.005-1.012)	0.692	r = 0.70	< 0.001*
BMI (per kg/m²)	1.674 (1.351-2.196)	0.425	r = 0.57	< 0.001*
Diastolic BP (per mmHg)	1.088 (1.032-1.158)	0.190	r = 0.35	0.004*
Systolic BP (per mmHg)	1.045 (1.016-1.078)	0.158	r = 0.35	0.003*
Hypertension (vs none)	5.92 (1.72-24.55)	0.131	V = 0.31	0.008*
Peripheral oedema (yes vs no)	3.27 (1.21-8.84)	0.098	V = 0.27	0.019*

## Discussion

In this hospital-based case-control study of adults with overt hypothyroidism, serum NT-proBNP was about fourfold higher and serum PDE4D about twofold lower than in age- and sex-matched euthyroid controls. Both differences were large and highly significant; the two markers moved in opposite directions, and they did so against a backdrop of higher BMI, blood pressure, and hypertension in the hypothyroid arm.

NT-proBNP in overt hypothyroidism

What makes the fourfold rise in NT-proBNP striking is its clinical context: none of the hypothyroid participants had overt heart failure on examination, and the design had already excluded known structural heart disease. The elevation therefore signals a subclinical, neurohormonal cardiac stress that routine assessment would miss. It also sits awkwardly with some of the earlier literature. Schultz et al. found NT-proBNP more than four times higher in hyperthyroid than in hypothyroid patients, and rising with free thyroxine [9]; Pakuła et al. saw no significant effect of either overt or subclinical hypothyroidism on NT-proBNP [10]; and a systematic review and meta-analysis reached much the same conclusion, that hyperthyroidism raises the peptide, while hypothyroidism lowers it or leaves it unchanged [19,20]. Those cohorts, though, were largely free of cardiovascular disease; ours was not. Our patients had long-standing disease (67.5% for at least five years), poor biochemical control (mean TSH: 25.75 mIU/L, with 47.5% poorly compliant), and a heavy cardiovascular-risk load: 35% were hypertensive, mean systolic pressure was 132 mmHg, mean BMI was 28.95 kg/m^2^, and 45% had oedema, every one of which independently raises NT-proBNP. In any hypothyroid patient, the measured value represents a balance between reduced thyroid-driven BNP transcription, which lowers it, and greater wall stress from higher afterload, expanded plasma volume, and impaired relaxation, which raises it [8,21,22]. In a cohort as burdened as ours, the haemodynamic drivers appear to have prevailed. A mean of 927 pg/mL would, in a symptomatic patient, prompt evaluation for heart failure, so finding it in an apparently ambulatory hypothyroid population is an argument for cardiovascular vigilance; it fits both the established prognostic weight of NT-proBNP [7] and the growing move toward multi-biomarker assessment of heart-failure risk [23].

Phosphodiesterase 4D in overt hypothyroidism

The fall in serum PDE4D is the less familiar and, we think, the more novel half of the picture. PDE4D is a cAMP-specific member of the PDE4 family; with PDE3, it handles much of the cAMP breakdown in the cardiomyocyte and helps set the cell's β-adrenergic responsiveness [12,13]. Since thyroid hormone upregulates several PDE isoforms [15,24,25], a hypothyroid state should lower PDE4D expression, slow cAMP hydrolysis, and let intracellular cAMP accumulate. PDE4D works on cAMP, whereas the natriuretic-peptide system signals through cGMP, so the two markers we chose sample complementary rather than overlapping arms of cyclic-nucleotide handling. Read that way, their opposite movement is most simply explained as broad dysregulation of cyclic-nucleotide signalling in hypothyroidism, not as one shared pathway [16]. In our hands, PDE4D separated hypothyroid from euthyroid participants better than any other variable measured, with a Nagelkerke pseudo-R^2^ of 0.884, ahead of even NT-proBNP. That number invites caution as much as enthusiasm: the assay measured one isoform by immunoassay rather than total phosphodiesterase activity, the sample was modest and single-centre, and the estimate needs replication before it can be trusted. Even so, it is a lead worth following. Circulating PDE4D deserves formal evaluation as a peripheral readout of thyroid-hormone action on the heart and vessels, and it might complement standard thyroid function tests when the clinical picture is ambiguous.

A reciprocal biomarker pattern

Two biologically distinct markers, neither part of the case definition, moved in opposite directions. That coherence makes it likelier that the pattern belongs to the hypothyroid state itself than to chance or to a single unmeasured confounder. The mechanism we propose, namely, reduced thyroid-hormone-driven expression of PDE4D and related isoforms with knock-on changes in intracellular cyclic-nucleotide handling, fits both the heart's recognised role as an endocrine organ and the known crosstalk between natriuretic-peptide and cyclic-nucleotide signalling in the myocardium [15,23]. Measuring the two analytes together may thus give a fuller account of the cardiovascular toll of hypothyroidism than either could alone.

Cardiovascular and metabolic correlates

The two biomarkers did not move in isolation. Around them, the hypothyroid arm showed a consistent cluster of subclinical cardiovascular strain: higher systolic and diastolic pressure, more than three times the prevalence of hypertension, and more frequent peripheral oedema. All of this is in keeping with what thyroid-hormone deficiency does to systemic vascular resistance and endothelial function, and with reports tying hypothyroidism to hypertension and to subclinical cardiac abnormalities [2,3,26-28]. The raised BMI likewise matches the obesogenic profile of the disease [29]. That extra adiposity is also a caveat, because obesity could itself drive part of the biomarker difference; our analyses were univariable, so we cannot yet separate an independent effect of thyroid status from one of body mass, and multivariable adjustment will be needed to tell them apart. Finally, that substantial biochemical hypothyroidism persisted despite universal levothyroxine prescription, set beside meta-analytic evidence that adequate replacement lowers TSH and improves the lipid profile [30], makes the case that dose optimisation and structured compliance support are cardiovascular measures as much as endocrine ones.

Strengths and limitations

This study has real strengths: an age- and sex-matched design with documented demographic comparability, the simultaneous measurement of two mechanistically complementary biomarkers by blinded laboratory staff, and a single statistical framework that reports effect sizes and regression estimates alongside p-values. Several limitations temper the interpretation. First, the cross-sectional case-control design precludes causal inference and cannot show whether the biomarker changes reverse with optimal treatment. Second, the sample of 80 participants at one tertiary-care centre limits precision and generalisability, and the cohort carried higher BMI, more hypertension, and longer disease duration than a community sample would. Third, no direct cardiac imaging (echocardiography, for instance) was done, so the inference of subclinical cardiac stress rests on biomarkers and haemodynamics rather than on structural confirmation. Fourth, we measured a single cAMP-specific isoform, PDE4D, as a protein concentration by immunoassay rather than total phosphodiesterase activity or other isoforms; the results therefore cannot be extended to overall phosphodiesterase activity, and circulating PDE4D need not track myocardial PDE4D expression or activity. Fifth, sampling was confined to a single time point, with no serial or longitudinal measurement to follow the change with treatment. Sixth, thyroid autoantibodies were not measured, leaving the aetiology of the hypothyroidism uncharacterised. Seventh, 47.5% of the cohort was poorly compliant with levothyroxine, so the group blended near-controlled with uncontrolled disease. Finally, because the analyses were primarily univariable and were not adjusted for BMI, blood pressure, age, or sex, residual confounding cannot be excluded. In particular, the higher BMI and blood pressure observed in the hypothyroid group may have independently influenced NT-proBNP levels; therefore, the independent effect of hypothyroidism on NT-proBNP cannot be established with certainty. Future prospective studies with larger sample sizes incorporating multivariable regression analyses are warranted to account for these potential confounders and validate our findings.

## Conclusions

In adults with long-standing, biochemically active overt hypothyroidism, serum NT-proBNP was about fourfold higher, and serum PDE4D about twofold lower than in age- and sex-matched euthyroid controls, and the two markers moved reciprocally. These shifts ran alongside higher blood pressure, BMI, and hypertension, which together sketch a state of subclinical cardiovascular compromise. Taken together, they make a case that patients with overt hypothyroidism may benefit from systematic cardiovascular surveillance and from tighter biochemical control, and they mark out serum PDE4D as a promising, hypothesis-generating candidate biomarker of thyroid-related cardiovascular stress. Because the study was single-centre and the analysis univariable, these observations need confirmation in larger, prospective, multivariable-adjusted work, spanning phosphodiesterase isoforms and testing against functional-activity assays, before serum PDE4D could count as an established marker.
